# Wear Performance of Ceramic-On-Metal Hip Bearings

**DOI:** 10.1371/journal.pone.0073252

**Published:** 2013-08-29

**Authors:** Jörn Reinders, Robert Sonntag, Christian Heisel, Tobias Reiner, Leo Vot, Jan Philippe Kretzer

**Affiliations:** Laboratory of Biomechanics and Implant Research, Department of Orthopedics and Traumatology, Heidelberg University Hospital, Heidelberg, Germany; Delft University of Technology (TUDelft), Netherlands

## Abstract

Ceramic-on-metal (CoM) bearings are considered to be a promising alternative to polyethylene-based bearings or hard-on-hard bearings (Ceramic-on-Ceramic (CoC) and Metal-on-Metal (MoM)). Although, CoM shows lower wear rates than MoM, in-vitro wear testing of CoM shows widely varying results. This may be related to limitations of wear-measuring methods. Therefore, the aim of this study was to improve the gravimetric measurement technique and to test wear behaviour of CoM bearings compared to CoC bearings. Level walking according to ISO-14242 was simulated for four CoM and four CoC bearings. Prior to simulation, errors in measurement of gravimetric wear were detected and improvements in measurement technique incorporated. The results showed no differences in mean wear rates between CoM and CoC bearings. However, the CoM bearings showed wear results over a wide range of wear performance. High reliability of wear results was recorded for the CoC bearings. Material transfer was observed on the ceramic heads of the CoM bearings. Therefore, for level walking a partial mixed or boundary lubrication has to be assumed for this type of bearing. CoM is a highly sensitive wear-couple. The reasons for the observed behaviour cannot be clarified from this study. Simulator studies have to be considered as an ideal loading condition. Therefore, high variations in wear rates as seen in this study, even at low levels, may have an adverse effect on the in-vivo wear behavior. Careful clinical use may be advisable until the reasons for the variation are fully clarified and understood.

## Introduction

Joint-related disorders of the hip such as persistent pain or functional disabilities can be successfully treated by total hip replacement (THR). Following register data, survival rates of approximately 90% after 10 years are reported [1]. However, the lifetime of an artificial joint is still limited and wear of the articulating surfaces remains a major limiting factor in this respect [2]. Nevertheless, expectations regarding quality of life and thus on implants are increasing [3]. The performance of implants therefore has to improve.

New-generation hard-on-hard bearings such as ceramic-on-ceramic (CoC) or metal-on-metal (MoM) are technically promising solutions for the wear problem. Currently, CoC is considered to be the gold-standard of a low-wearing bearing in THR with a decreased incidence of osteolysis [4]. However, breakage (rare incidence of 0-2% depending on the ceramics used [5–7]) and squeaking (incidence 0.5-20.9% [5,8]) of ceramic components are still a concern. For MoM, laboratory studies have shown low wear [9]. However, concerns exist regarding interaction of metal wear products with the immune system, which might take even place at low wear volumes [10]. In this context, cases of high-wear with local effects (metallosis, necrotic tissue, pseudotumours) [11,12] up to those showing massive-wear with systemic effects [12] have been reported and these have alarmed the orthopaedic community.

THR using a metallic liner and a ceramic head (CoM) has recently been introduced clinically to meet the aforementioned challenges. It is not known how many CoM bearings have been implanted to date. One study states that at least 10,000 CoM bearings have been implanted [13], but it is not clear how this number was derived. In fact, although CoM bearings are used clinically there is little knowledge about their in-vivo performance [14]. Some case reports have shown tremendous wear of CoM [15,16], but in those cases the technical design differed from that of modern CoM bearings. Keeping this in mind, simulator studies may be an important tool for acquiring knowledge of possible CoM-related risks.

Although some laboratory studies have shown low wear rates of CoM [17–23], there is a high variance between the results of different research groups. Wear rates from published studies differ by a factor of 150 [17,20].

Possible reasons for this discrepancy are:

- First, CoM is a highly sensitive wear-couple with widely ranging wear performance.
- Second, the wear rate is generally low. Differences are due to limitations of the methods used to measure wear (mostly gravimetric).

The aim of this study was therefore to test the wear behaviour of CoM compared with CoC. To increase the validity of the results this study focused on improvements in the gravimetric measurements technique. The results are supported by continuous screening of the cobalt and chromium release, as important parameters for the clinical use.

## Material and Methods

### Simulator-study

For wear testing, four pairs of CoC bearings (Biolox®Delta, Ceramtec) and four pairs of CoM bearings (CoMplete, DePuy) with a nominal diameter of 36 mm were used. Wear tests were run on a single-station hip joint simulator (858 Mini Bionix II system; MTS Systems, Minnesota, USA). Level walking according to ISO 14242-1 was simulated. This includes a dynamic load up to 3 kN, a maximum flexion of 25° to 18° of extension, an adduction of 7° to 4° of abduction and an internal rotation of 2° to 10° of external rotation. Simulation was run to a total of 2.4 million cycles corresponding to approximately 1 year of use in an active patient [24]. Bovine serum with a protein content of 30 g/L was used as a substitute for synovial fluid. The simulation was interrupted every 200,000 cycles to clean and weigh the components. Every 400,000 cycles the bovine serum was completely changed.

Prior to simulation, CoC and CoM components were measured using a coordinate measuring machine (MarVision MS 222, Mahr, Göttingen, Germany; accuracy: ± 2.3 µm). Diameter and deviation of roundness of the heads and cups of both groups were determined. Subsequently the components were matched in order to achieve comparable clearances within both groups.

**Figure 1:**
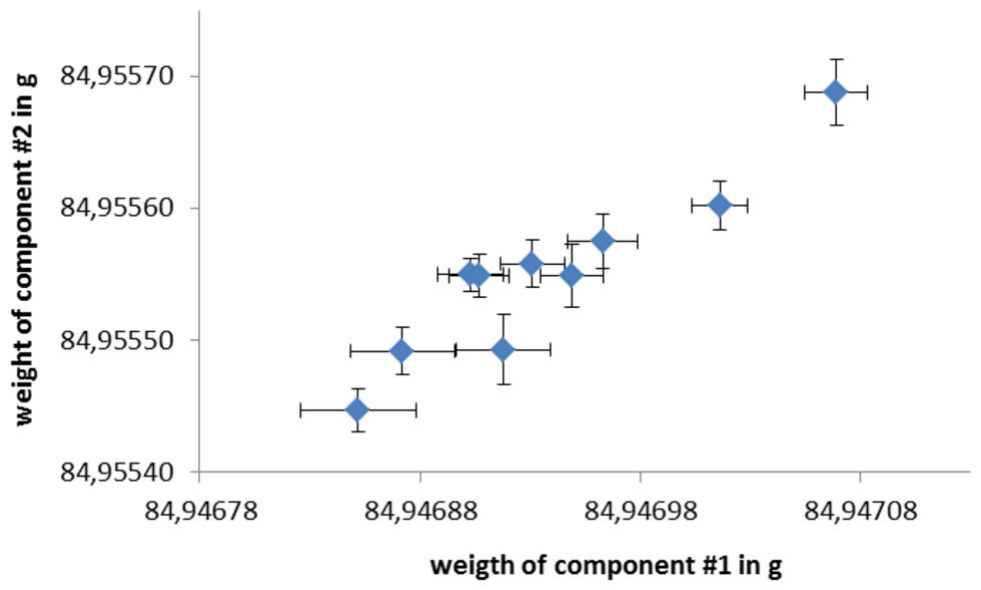
Relationship between weight determinations of two ceramic heads.

### Gravimetric repeatability, stability & disturbance reduction

For wear measurements, a high-precision balance (Genius ME 235S, Sartorius, Göttingen, Germany) with a specified repetitive accuracy of 25 µg was used. The repetitive accuracy specifies the accuracy of the measurement in the presence of identical physical conditions [25]. However, disturbances due to minimal changes of temperature, humidity and atmospheric pressure cannot be excluded even in a temperature-controlled room.

To characterise the system of gravimetric measurement used, two parameters were analysed: 1) The **repeatability** of a measurement system specifies variations in measurement under identical conditions (within one series of measurement) 2) The **stability** of a measurement system specifies differences of the mean value within a defined time interval (differences between measurement series).

Therefore the components (ceramic head, ceramic liner, metal liner) were measured in an own measurement series prior to simulation. Every series consisted of 10 measurement intervals during a time period of 30 days (stability analysis). Each of these 10 measurement intervals consisted of a gravimetric analysis (repeatability analysis) which was repeated for 12 times.

Determination of possible disturbances provides the opportunity for correction of measurement results. It was hypothesized that disturbing factors would act in the same way on identical measuring objects. To test this hypothesis, we performed an additional series of measurements on which two nearly identical (deviations only caused by production) components (two ceramic heads, two ceramic liners, two metal liners) were measured as described above.

### Gravimetric analysis

The components were cleaned using mild detergents, ultrasonic baths and isopropanol in a repeating sequence. After the cleaning process the implants were vacuum-dried and tempered for at least 12 hours in a temperature-controlled precision measurement room. Each measurement was repeated 12 times. Calibrated control weights (Zwiebel, Saverne Cedex, France) were used as an additional reference during measurements. Volumetric wear was calculated based on the density of the ceramic (ρ_c_ = 4.37 mg/mm^3^) and the metal (ρ_m_ = 8.3 mg/mm^3^) components.

**Table 1:** Gravimetrical determination of stability and repeatability of unused components.

	**Stability**				**Repeatability**
Component	Mean	Min.	Max.	Deviation	SD max.
Ceramic head	84.85418 g	84.85395 g	84.85434 g	390 µg	≤ 0.00002
Ceramic cup	60.96731 g	60.96713 g	60.96743 g	300 µg	≤ 0.00002
Metal cup	115.27250 g	115.27243 g	115.27253 g	100 µg	≤ 0.00002

### Metal ion analysis

The simulations were run in a metal-free, low-contamination environment [26,27]. Fluid samples were taken every 200,000 cycles under clean room conditions. Cobalt and chromium content were analysed using high-resolution, inductively coupled mass spectroscopy (Element2; Thermo, Fisher Scientific, Bremen, Germany) according to previous published methods [26,27].

**Figure 2:**
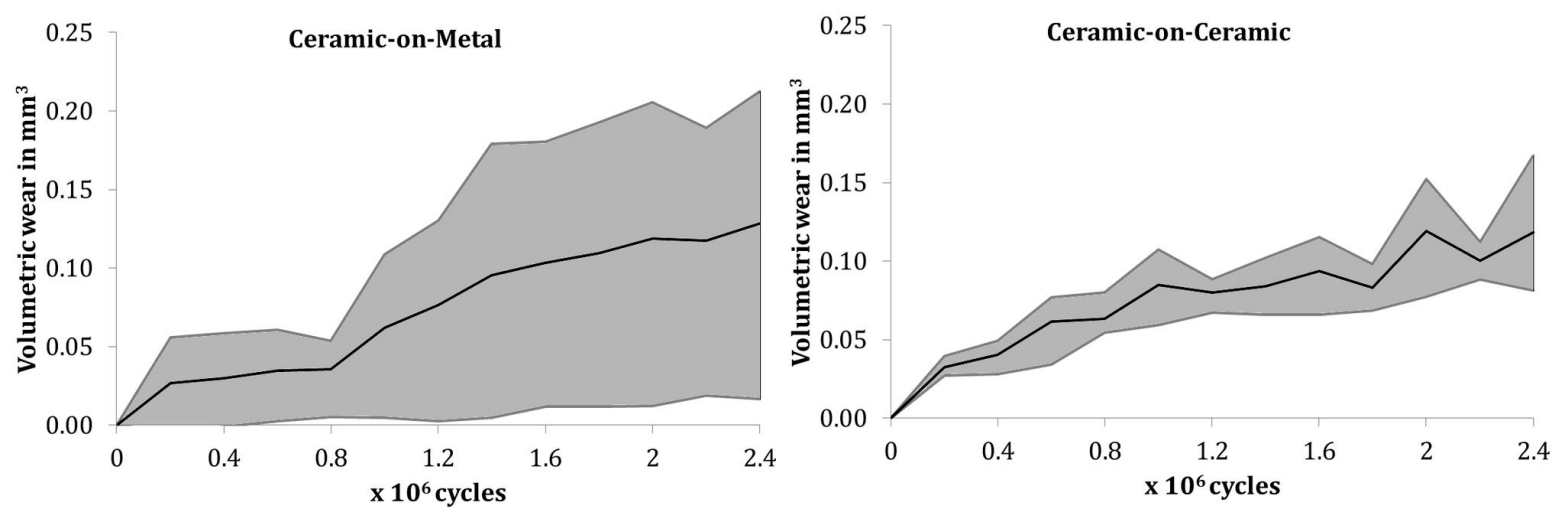
Volumetric wear of tested bearings (left: CoM; right: CoC). Grey area: mean and range of lowest and highest wear couple.

### Statistics

The wear rates of both bearings were compared using Student’s t-test for two independent parametric samples with a level of significance set at p<0.05 (SPSS 16.0.1, SPSS Inc., Chicago, IL). All data are presented as mean ± standard deviation (SD).

## Results

### Gravimetric stability & repeatability, stability & disturbance reduction

Table 1 shows the results for stability and repeatability. High repeatability (deviation within one measurement) was found for every component. Stability, as a parameter for time-dependent differences in measurement results, shows differences of up to 390 µg for the ceramic head, but only 100 µg for the metal cup. Deviations can be defined as physical disturbances (changes in atmospheric pressure, temperature and humidity) [25] and these were found to be in wear-relevant ranges.

Significant correlations were observed between identical measuring objects for all pairings (Table 2, Figure 1). Generally, ceramics showed a better statistical relationship than metal cups did. However, the hypothesis that systematic disturbances can be detected by measuring nearly identical measuring objects was confirmed.

**Table 2:** Statistical relationship between two identical measuring objects.

	Correlation coefficient	p value
Ceramic head	0.937	p ≤ 0.001
Ceramic cup	0.930	p ≤ 0.001
Metal cup	0.772	p = 0.009

To compensate for these disturbances during wear simulation, physically and geometrically identical components (deviations ≤ 1%) were measured in parallel; based on this, a correction of the gravimetric results of the wear components was performed.

### Simulation and gravimetric analysis

Clearance was lower for the CoC bearings (23.8 ± 3.1µm) than for CoM bearings (51.1 ± 2.0µm). Only small differences in deviations of roundness were observed (CoC: 5.49 ± 1.28µm; CoM: 4.84 ± 2.0µm)

No differences in wear rates (overall, running-in, steady state) were found between CoC and CoM (Table 3, Figure 2). However, wear rates of CoM bearings showed higher variations than CoC bearings. Overall the lowest and highest wear rates were observed for CoM bearings. The lowest wear rate for CoM was 0.017 mm^3^/10^6^ cycles compared to 0.081 mm^3^/10^6^ cycles for CoC. The highest wear rate of CoM was 0.212 mm^3^/10^6^ cycles compared to 0.167 mm^3^/10^6^ cycles for CoC.

**Table 3:** Mean, SD and range of wear rates of CoC and CoM bearings.

	Running-in wear in mm3/10^6^ cycles	Steady-state wear in mm3/10^6^ cycles	Overall wear in mm3/10^6^ cycles
CoC	0.163 ± 0.026 (0.137-0.199)	0.038 ± 0.012 (0.027-0.055)	0.118 ± 0.036 (0.081-0.167)
CoM	0.134 ± 0.134 (-0.019-0.279)	0.053 ± 0.038 (0.009-0.090)	0.129 ± 0.096 (0.017-0.212)
p value	0.697	0.571	0.851

**Figure 3:**
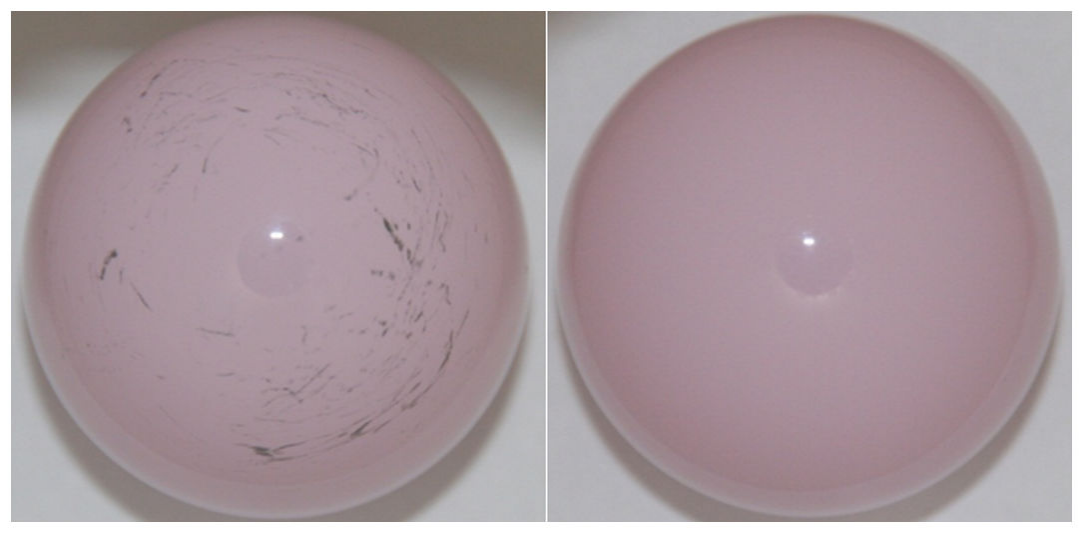
Head of CoM bearing (left) and CoC bearing (right).

Wear of the CoC components was evenly distributed between heads (43.1 ± 9.2%) and inserts (56.9 ± 9.2%). For the CoM components wear was observed mainly on the metal inserts (97.2 ± 3.5%). Only marginal wear was observed on the ceramic heads (2.8 ± 3.5%).

Ceramic heads from the CoM bearings showed visible signs of metallic transfer in the form of stripes. For the ceramic heads of CoM bearings, the subjective amount of material transfer was higher for bearings with higher wear rates compared to the low-wearing CoM bearings. In comparison, no visible traces were found on the CoC heads (Figure 3).

### Metal ion analysis

**Figure 4:**
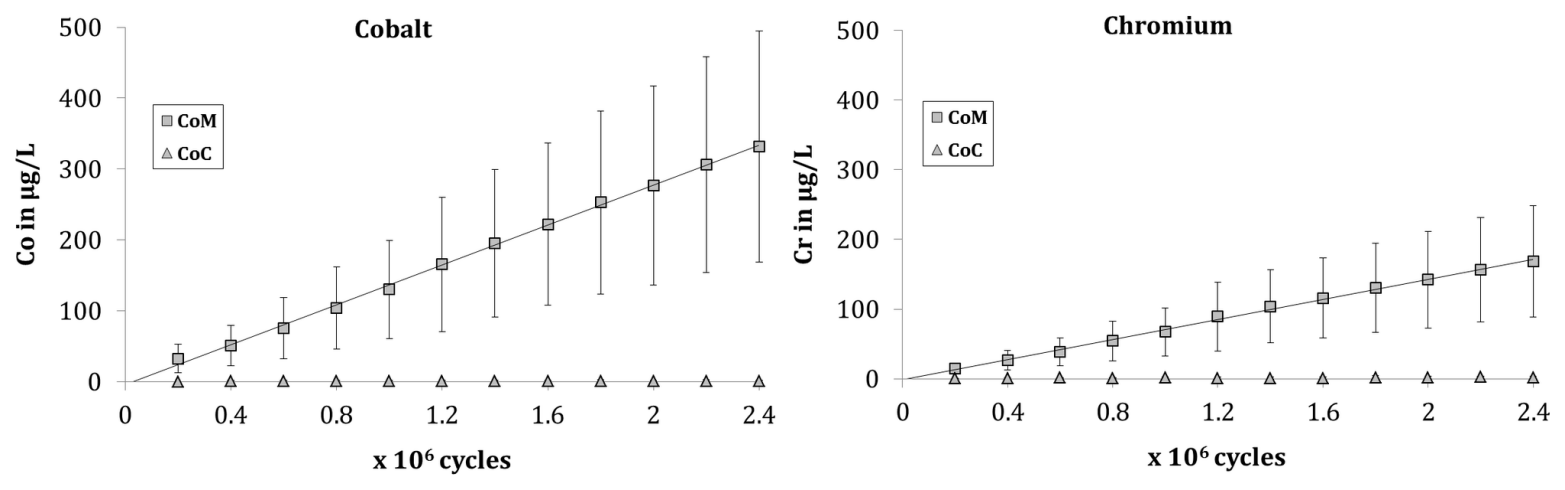
Cumulative release of cobalt (left) and chromium (right).

Results of ion-measurements are shown in Figure 4 for cobalt and chromium. As expected no ion-release of cobalt and chromium was observed for the CoC bearings. Additionally, these measurements confirm that no contamination occurred due to the simulator environment.

Measurements of the CoM bearings showed a continuous increase in the release of chromium and cobalt. By the end of the test 168 µg/L chromium and 331 µg/L cobalt had been released. 

## Discussion

Following successful improvement of the gravimetric measurement technique, this study compared the wear behaviour of the new CoM bearings to the gold-standard of CoC bearings. It has been shown that deviations in measurements (instability) occur over time, probably due to minimal changes in ambient conditions (intrinsic disturbances) [25]. These deviations were in wear-relevant ranges. By using equivalent reference heads and cups these disturbances can be reduced and the validity of measurement increased.

However, gravimetric measurements still exhibit limitations. A complete dismounting/mounting and cleaning are necessary. This might change the tribological system:

- Wear influencing layers (e.g. tribolayer, phophatlayer etc.) may be removed [27,28].
- With every mounting, components are macroscopically exactly aligned. Nevertheless, a correct alignment on a micron scale is technically difficult to realise. A slight component misalignment with an impact on wear cannot be excluded.
- Incomplete cleaning and possible contamination cannot be excluded

Despite these limitations, gravimetric measurements are a highly important tool for determining wear. Additional information on the wear performance of implants can be provided by ion analytic measurements. Regarding ion exposure, a more continuous increase was detected without the variations that still exist in gravimetric measurements.

The mean wear of CoM and CoC bearings was comparable. However, a high variation was observed for CoM. The ratio between minimal and maximal wear was 12 for CoM and 2 for CoC.

**Figure 5:**
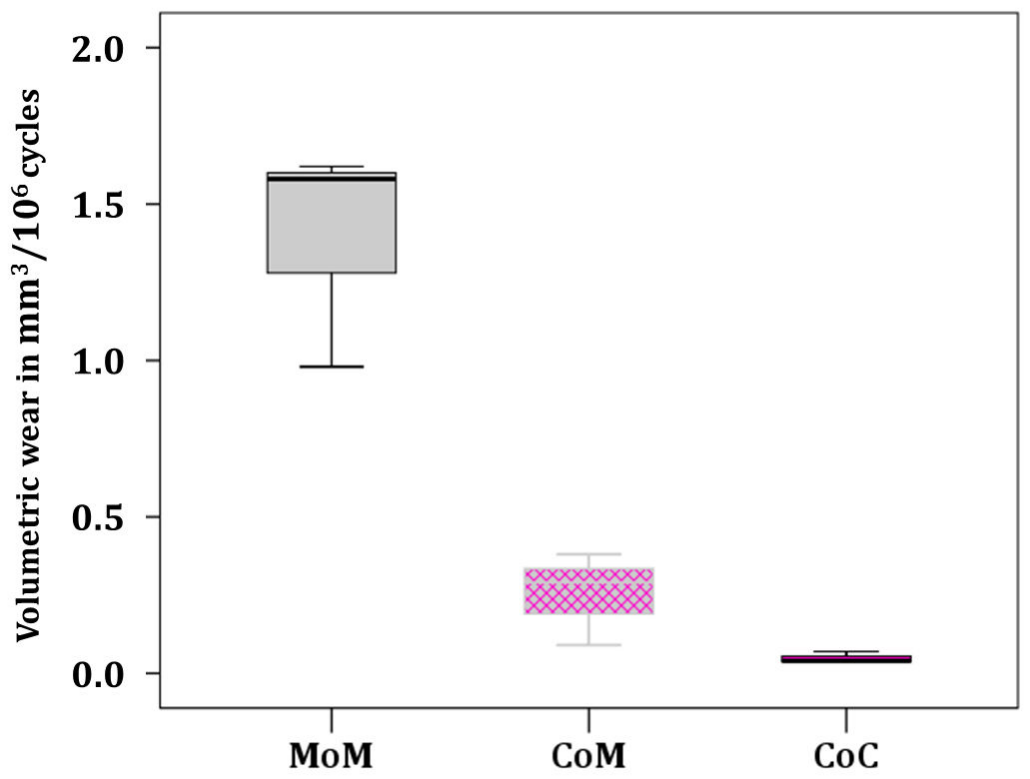
Literature data of CoM studies including control bearings (MoM and CoC)

In Figure 5 a box-and-whisker diagram shows differences between literature data regarding CoM simulator studies and the used control bearings (CoC & MoM). CoC bearings consistently show only small mean variations among different research groups. Wear results are also consistently low. These observations were confirmed in this study. The literature data confirm a characteristically wider range of wear for CoM than for CoC, and show that MoM bearings exhibit wear at a higher level and with greater variation than CoM bearings [17–20,22,23].

Lubrication is assumed to be a key factor in the tribological performance of hard-on-hard bearings [29,30]. Simplified and theoretical models provide insights into possible fluid-film thicknesses and the predominant lubrication regimes. These models are based on material characteristics (clearance, roundness, roughness, material elasticity etc.), environmental characteristics (fluid viscosity) and activity characteristics (kinematics and kinetics) [31]. Regarding level walking (as simulated according to ISO 14242) these models indicate a mixed to fluid-film lubrication for MoM bearings and fluid-film lubrication for CoC bearings [32]. Although these models still display limitations [33,34], they have attained a high level of complexity and offer the possibility to predict in-vivo lubrication to a certain extent. In a recent study, Meng et al. [35] analysed the lubrication mode of hard-on-hard bearings including CoM bearings. They reported fluid-film lubrication for CoC during the whole gait cycle. A slightly inferior lubrication mode was found for CoM, with 80% of the gait cycle operating in the fluid-film mode. In the present study observation of metal transfer to the ceramic heads indicates a contact between both bearing partners.

Only a few studies have been published on the clinical performance of CoM bearings [21,23,36]. Two of these [21,23] were follow-up studies from the same centre with different patient collectives, reporting results after 3 and 12 months [21] and 6 months [23] postoperatively. The median ion release of cobalt and chromium after 6 months showed no significant differences between CoM and MoM [23]. Significantly reduced median cobalt and chromium release was observed after 12 months [21]. Only a small number of patients were compared for CoM and MoM. Additionally, a relatively high number of outliers were excluded for both CoM (1 of 7 = 17% [23] and 4 of 22 = 18% [21]) and for MoM (1 of 7 = 17% [23] and 4 of 19 = 21% [21]). In a double-blinded randomized study, Schouten et al. [36] compared chromium and cobalt release in patients treated with CoM (n = 41) and MoM (n = 36) bearings and found no differences, after 6 and 12 months. At present, therefore, clinical follow-up shows no clear advantage of CoM bearings.

The clinical relevance of simulator studies is limited. The main limitation is the replication of one loading condition (walking) in a standardised system with matched bearings. In-vivo, multiple loading conditions occur, possibly resulting in different wear behaviours. Moreover, wear performance is potentially influenced by individual factors (alignment, synovial fluid, ligamental situation etc.).

In this context the introduction of microseparation [37] in simulator testing has been found to produce clinically relevant wear characteristics. To date, three simulator studies [23,38,39] have reported on the wear behaviour of CoM bearings under these tribologically adverse conditions. Lower wear than in MoM bearings is described in two of these studies [23,38]. In the third [39], CoM was analysed without a control group: higher absolute wear of CoM bearings was found than in the other two studies. The standard deviations [38,39] of the wear results were high, indicating considerable variance of wear.

We have not yet attained a full understanding of these complex in-vivo and in-vitro relationships. The excellent wear results of MoM in simulator studies have been confirmed only to a limited extent in the clinical situation. Some clinical trials have shown excellent long-term wear performance of MoM [14], but increasing numbers of studies have reported catastrophic results of MoM [12]. The reasons for this discrepancy have not yet been fully elucidated. With regard to the variation in wear behaviour of CoM, clinical follow-up studies are required to establish which patients may benefit from CoM bearings.

## Conclusion

In this study CoM exhibited mean wear results as good as those for CoC. However, CoM is a highly sensitive wear-couple. The individual CoM bearings exhibit considerable variation in wear. Regarding the lubrication of CoM bearings, contact between bearing surfaces has been demonstrated and therefore a partial mixed or boundary lubrication has to be assumed. In contrast, CoC shows consistently low wear rates and therefore greater reliability. The reasons for these differences in behaviour require further clarification. Simulator studies such as ours have to be considered to provide ideal loading conditions with respect to matching of components, loading and alignment. Therefore, the high variation of wear in CoM seen in this study, even at low levels, may indicate potential problems with CoM bearings in-vivo. Caution in clinical use may be advisable until the reasons for the variations in wear are understood. 
